# PD-Loma: a cancer entity with a shared sensitivity to the PD-1/PD-L1 pathway blockade

**DOI:** 10.1038/s41416-018-0294-4

**Published:** 2018-11-09

**Authors:** Laure Hirsch, Laurence Zitvogel, Alexander Eggermont, Aurelien Marabelle

**Affiliations:** 1 Département d’Innovation Thérapeutique et d’Essais Précoces, Gustave Roussy, Université Paris-Saclay, Villejuif, F-94805 France; 2 Gustave Roussy, Université Paris-Saclay, INSERM U1015, Villejuif, F-94805 France; 3 Gustave Roussy, Université Paris-Saclay, Villejuif, F-94805 France

## Abstract

Clinical trials have now identified over 30 cancer histotypes with sensitivity to anti-PD-(L)1 therapies. It is the first time in oncology that a class of drugs has demonstrated such a wide spectrum of activity in monotherapy. This subgroup of cancers (‘PD-Lomas’) is driving the clinical research strategies for the next generation of combination immunotherapy.

## Main

Conventional cytotoxic chemotherapies, which often do not induce immunogenic cell death, display moderate efficiency and are associated with major adverse side effects in numerous advanced cancers. The paradigm of personalised medicine with tumour-targeted therapies has markedly increased our understanding of cancer cell biology, and can deliver impressive clinical results . However, these improvements are generally limited to a subset of patients with a given cancer type, and the benefits are mostly in terms of progression-free survival rather than overall long-term survival. The development of new anti-cancer therapies to extend the patient’s overall survival and provide a better quality of life represents a major challenge.

An improved understanding of the pathways involved in the immune escape of cancer cells has allowed us to identify the important role of immunosuppressive checkpoint molecules, such as programmed cell death 1 receptor (PD-1) and its ligand programmed cell death ligand 1 (PD-L1). Under physiological conditions, the interaction of PD-1 with its ligand PD-L1 results in T cell immune suppression. This immune checkpoint pathway is used by ‘self’ cells to prevent autoimmunity; however, it can be hijacked by cancer cells to escape from anti-tumour immunity. PD-1 is highly expressed by cancer-specific T cells infiltrating into tumours. Its ligand, PD-L1, is upregulated on a variety of cells in the tumour microenvironment, including cancer cells, stromal cells, and immune cells, in response to T cell-released gamma interferon. Antagonistic monoclonal antibodies targeted at PD-1 (nivolumab [N], pembrolizumab [P]) or PD-L1 (atezolizumab [At], durvalumab [D] and avelumab [Av]) allow restoration of the anti-tumour T cell functions and can generate durable objective tumour responses in patients.

## Addressing a universal feature of cancer development

Numerous phase I studies have shown durable anti-tumour activity at least in few patients treated with anti-PD-(L)1 monotherapies for various solid tumours. This long-term efficacy has translated into overall survival benefits in several phase II and III studies. Demonstration of their clinical efficacy has led to FDA approving the use of anti-PD-(L)1 monotherapies for melanoma [N;P], squamous and non-squamous non-small-cell lung cancers (NSCLC) [N;P;At], renal cell carcinoma [N], head and neck cancer [P;N], classical Hodgkin lymphoma [P;N], urothelial carcinoma [P;N;D;At], advanced gastric cancer [P], hepatocellular carcinoma [N], microsatellite instability-high (MSI) cancers [P], and Merkel cell carcinoma [Av] in the United States. While conventional strategies require a specific chemotherapy regimen for each type of cancer, anti-PD-L1 therapies have demonstrated efficacy when used as monotherapy for malignancies as different as squamous cell carcinomas and adenocarcinomas (e.g. in NSCLC), melanomas, lymphomas, sarcomas, small cell carcinomas or neuroendocrine tumours (e.g. Merkel cell carcinoma). This efficiency demonstrates that there are common immunosuppressive pathways across malignancies, and that therapeutic strategies against a universal feature of cancers, such as the immune component, could potentially be developed independently of their histotype.

**Fig. 1:**
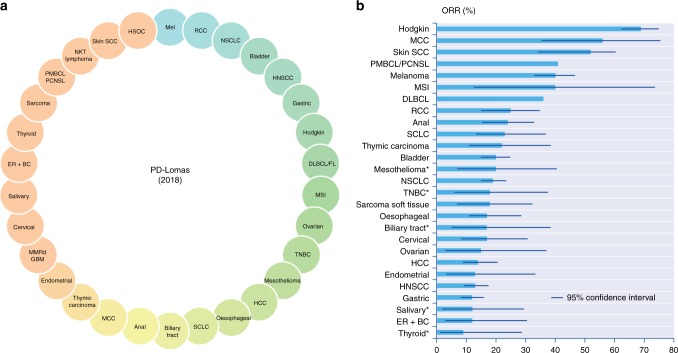
PD-Lomas: cancers with known sensitivity for anti-PD-(L)1 monotherapies. **a** Tumour types where some clinical activity of anti-PD-(L)1 has been reported. *ORR for cancer histotypes with PD-L1 positivity, defined by expression in ≥1% of tumour cells by immunohistochemistry. **b** Objective response rates per cancer histotype upon anti-PD-(L)1 monotherapy. Mel melanoma; RCC renal cell carcinoma; NSCLC non-small-cell lung cancer; HNSCC head and neck squamous cell carcinoma; DLBCL diffuse large B-cell lymphoma; FL follicular lymphoma; MSI CRC microsatellite instability-high colorectal cancer; TNBC triple negative breast cancer; HCC hepatocellular cancer; SCLC small-cell lung cancer; MCC Merkel cell carcinoma; MMRd GBM mismatch repair-deficient glioblastoma; ER+BC oestrogen receptor-positive breast cancer; PMBCL primary mediastinal B-cell lymphoma; PCNSL primary central nervous system lymphoma; NKT lymphoma natural Killer/T cell lymphoma; SCC squamous cell carcinoma; HSOC high-grade serous ovarian cancer; ORR objective response rate.

In the current era, in which many novel immunotherapies are being developed in the clinic and strategic choices have to be made in terms of the cancer indications, it may be useful to designate cancers with known sensitivity to anti-PD-(L)1 monotherapy as opposed to designating cancers with no sensitivity to immunotherapy (e.g. microsatellite stable [MSS] colorectal carcinoma, pancreatic cancers, sarcoma, glioblastoma, prostate cancers, among others). Cancer histotypes with at least a 10% objective response rate (ORR) upon anti-PD-(L)1 monotherapy could be categorised as a ‘PD-Loma’ (Fig. 1a).

There is heterogeneity in response rates among PD-Lomas. Melanoma, skin SCC, Merkel cell, MSI cancers and Hodgkin lymphoma are among the best responders to anti-PD-(L)1. The ORRs for melanoma and Hodgkin lymphoma are in the range of 33–40% and 66–69%, respectively.^1–4^ Patients with MSI colorectal cancer or skin SCC have ORRs of 40% and 52%, respectively.^5,6^ ORRs range between 10 and 20% for other tumour types. For example, anti-PD-(L)1 as second-line therapy in NSCLC has an ORR range of 15–20%; however, this increases to 45% when at least 50% of tumour cells express PD-L1.^7,8^ Renal cell carcinoma (ORR = 25% with N),^9^ head and neck squamous cell carcinoma (ORR = 18% with P)^10^ and ovarian cancers (ORR = 15% with N)^11^ are other examples of cancers with such sensitivity to anti-PD-(L)1 monotherapy in patients with 2+ previous lines of treatment (Fig. 1b). Interestingly, ORRs with anti-PD-(L)1 therapy are always lower than those for anti-PD-1, but this does not translate into significant differences in OS.^12^ In contrast, concurrent therapy with nivolumab and ipilimumab (an antibody against cytotoxic T-lymphocyte-associated antigen 4 [CTLA-4]) allows for ORR improvements,^13,14^ but with increased toxicity. In the near future, several new immunotherapy combinations are expected to improve the response rates in PD-Lomas.

## Challenges for the treatment of PD-Lomas

Patients do not respond to immunotherapy with the same clinical and radiological patterns as for chemotherapy or tumour-targeted therapies. Progression-free survival (PFS) under immunotherapy does not seem to be any better than with chemotherapy in many studies, although there are clear benefits regarding the overall survival. This can be explained by the fact that patients responding to immunotherapy have prolonged control of their disease, compared to that usually seen with standard treatments. This durability of response is due to three factors. First, anti-PD-(L)1 therapies supposedly generate a polyclonal T cell response against cancer antigens, which should help address the heterogeneity of cancer cells (as opposed to mono-targeted therapies). Second, T cell responses can have memory features that should also contribute to the durability of the anti-tumour immunity. Third, T cells can cross the blood–brain barrier (as opposed to drugs such as antibodies), and anti-tumour immunity generated upon anti-PD-(L)1 therapy might better protect against CNS progressions and/or relapses.

PFS survival curves, although commonly used for conventional treatment modalities, are not an ideal endpoint for immunotherapy clinical trials. Patients who do not respond to immunotherapy can progress as fast as with a conventional regimen or even faster, and PFS curves mostly capture events from RECIST progressors at the beginning of the curve. Indeed, hyperprogressive disease has also been reported upon anti-PD-(L)1 therapy. This paradoxical effect is not fully captured in clinical trials with late first CT-scan evaluation and non-reported clinical progressors;^15^ earlier (e.g. 1-month) CT scans might be necessary to objectively capture these initial ‘clinical progressions’. Furthermore, upon initiation of anti-PD-(L)1 therapy and the subsequent promotion of T cell recruitment/expansion, pre-existing tumour lesions may initially increase and new radiological lesions may transiently appear before obtaining a stable or partial/complete response. These pseudo-progressions and/or mixed responses are classified as progressions per RECIST 1.1 criteria. Therefore, it seems necessary to use dedicated radiological criteria, such as immune Response Evaluation Criteria in Solid Tumours (iRECIST), to assess these atypical tumour responses and to capture the spectrum of clinical benefits of immune-targeted therapies.^16^ Moreover, PFS curves do not correlate with the overall survival benefits of immunotherapies that are explained by long-term responses, and possibly sensitisation of the tumour to the next line of chemotherapy.

Besides the refinement of our clinical assessments, the identification of predictive biomarkers would allow targeted prescription of these drugs to patients who have the potential to benefit, avoiding the exposure of patients to unnecessary toxicity when there are no chances of efficacy. Several cancer-related (tumour mutation burden, PD-L1 expression, HLA expression) or host-related (T cell infiltrates, IDO expression, gamma interferon signature, relative neutrophil counts and gut microbiome composition) biomarkers have been proposed for analysis at baseline, because they correlate with the survival of patients treated with anti-PD-(L)1 therapies. Notably, 15+ of these PD-Lomas reside in the mucosae, raising the question of the surrounding microbiota's impact on the potential cross-reactivity with tumour neoantigens, and the potential detrimental impact of systemic antibiotics.^17^ Unfortunately, the low level of sensitivity and specificity of these biomarkers currently make them irrelevant at the individual patient level, but combining several of these markers may help to increase their predictive ability (e.g. selecting patients with both high tumour mutation burden and high tumour T cell infiltrates). Alternatively, we could aim to identify biomarkers immediately following initiation of anti-PD-(L)1 monotherapy, to identify early on if the patient will eventually progress or respond to therapy. Biomarkers with good negative predictive value would also be useful, by allowing us to avoid initiating immunotherapy in patients with no chance of response. This approach would be particularly relevant in the context of anti-PD-(L)1 therapy, because most patients with stable disease also significantly benefit from the treatment and contribute to the overall survival benefit. Such a pre-emptive strategy would allow us to build a therapeutic stratification of patients, and to quickly change the treatment modality for patients who are refractory to anti-PD-(L)1 monotherapy.

A large number of anti-PD-(L)1 combination trials are currently ongoing. A frequent criticism claims that these trials are performed without any scientific rationale; however, most of these combinations have a strong scientific background developed over the past 15 years, demonstrating combinatorial synergies with these drugs at the pre-clinical level. This aggressive clinical development is also supported by the broad spectrum of activity of anti-PD-(L)1 therapies, with the common aim of overcoming resistance to monotherapy and increasing the level of clinical benefit. A significant part of the future of immunotherapy development will thus be empirical; some combinations will translate well from mice to humans, some will not. However, we can expect that some cross-fertilisation will subsequently happen, and that synergistic combinations seen in one indication could then be successfully tested in several other cancers.
